# Effect of *GSTA1* Variants on Busulfan-Based Conditioning Regimen Prior to Allogenic Hematopoietic Stem-Cell Transplantation in Pediatric Asians

**DOI:** 10.3390/pharmaceutics14020401

**Published:** 2022-02-11

**Authors:** Ai-Hoc Nguyen, Mohitosh Biswas, Apichaya Puangpetch, Santirhat Prommas, Samart Pakakasama, Usanarat Anurathapan, Jiratha Rachanakul, Rattanaporn Sukprasong, Nutthan Nuntharadtanaphong, Nutcha Jongjitsook, Suradej Hongeng, Chonlaphat Sukasem

**Affiliations:** 1 Division of Pharmacogenomics and Personalized Medicine, Department of Pathology, Faculty of Medicine Ramathibodi Hospital, Mahidol University, Bangkok 10400, Thailand; aihoc050994@gmail.com (A.-H.N.); biswas_07pharm@ru.ac.bd (M.B.); apichaya.pua@mahidol.ac.th (A.P.); santirhat.pro@mahidol.ac.th (S.P.); jiratha.rac@mahidol.edu (J.R.); rattanaporn.suk@mahidol.ac.th (R.S.); nutthan.nun@mahidol.ac.th (N.N.); pearnutcha_1997@hotmail.com (N.J.); 2 Laboratory for Pharmacogenomics, Somdech Phra Debaratana Medical Center (SDMC), Ramathibodi Hospital, Bangkok 10400, Thailand; 3 Department of Pharmacy, Faculty of Science, University of Rajshahi, Rajshahi 6205, Bangladesh; 4 Division of Hematology-Oncology, Department of Pediatrics, Faculty of Medicine Ramathibodi Hospital, Mahidol University, Bangkok 10400, Thailand; samart.pak@mahidol.ac.th (S.P.); usanarat.anu@mahidol.ac.th (U.A.); suradej.hon@mahidol.ac.th (S.H.); 5 Division of Pharmacogenomics and Precision Medicine, The Preventive Genomics & Family Check-Up Services Center, Bumrungrad International Hospital, Bangkok 10400, Thailand; 6 MRC Centre for Drug Safety Science, Department of Pharmacology and Therapeutics, Institute of Systems, Molecular and Integrative Biology, University of Liverpool, Liverpool L69 3GL, UK

**Keywords:** busulfan, hematopoietic stem-cell transplantation pharmacokinetics, GST genetic polymorphisms, pediatric Asians

## Abstract

Busulfan is widely used as a chemotherapy treatment before hematopoietic stem-cell transplantation (HSCT). However, the response of busulfan is highly variable and unpredictable, whereby the pharmacogenetic interference of glutathione *S*-transferase (GST) has strong evidence in Caucasians and some adult Asians but not in pediatric Asian patients. This study was aimed at investigating the associations of *GST* genetic polymorphisms with variations in the pharmacokinetic (PK) properties of busulfan in pediatric Asian patients. This retrospective cohort study recruited 92 pediatric patients. The polymorphism of *GSTA1* was genotyped by Sanger sequencing, and *GSTM1* and *GSTP1* were genotyped by real-time PCR. Drug concentration and PK estimation were identified using an LC-MS/MS method and a noncompartmental model. Statistical analysis was performed by R software. Out of 92 patients, 48 (53%) were males, the mean age was 8.4 ± 5.12 years old, and the average weight was 26.52 ± 14.75 kg. The allele frequencies of GSTA1*B and of *GSTM1* and GSTP1* deletions were 16.9%, 68.5%, and 21.2%, respectively. Patients with GSTA1*B had a statistically significant impact on the PK of busulfan, whereas those with *GSTM1* and *GSTP1* did not (*p* > 0.05). The carriers of GSTA1*B showed a significant difference compared to noncarriers in terms of t_1/2_ (for first dose: 161.9 vs. 134.3 min, *p* = 0.0016; for second dose: 156.1 vs. 129.8, *p* = 0.012), CL (88.74 vs. 124.23 mL/min, *p* = 0.0089), C_max_ (4232.6 vs. 3675.5 ng/mL, *p* = 0.0021), and AUC (5310.6 vs. 4177.1 µM/min, *p* = 0.00033). The augmentation of AUC was around 27.1% in patients carrying the GSTA1*B variant. The *GSTA1* polymorphism was significantly associated with variations of the pharmacokinetic properties of busulfan treatment in pediatric Asian patients.

## 1. Introduction

Busulfan is a bifunctional alkylating agent, widely used as chemotherapy treatment before hematopoietic stem-cell transplantation (HSCT). High exposure of busulfan is needed to destroy cancer cells, but it can also destroy normal hematopoietic cells. Subsequently, HSCT is used to “rescue” the patients from the side effects of chemotherapy [1]. Busulfan serves not only as a cornerstone for the success of this process, but also as a replacement for total body irradiation (TBI) of the conditioning regimen before HSCT [2,3,4].

The variation of busulfan’s response is highly unpredictable in terms of both pharmacokinetic (PK) and pharmacodynamic (PD) aspects. Exceeding the therapeutic range can cause graft-versus-host disease (GvHD), sinusoidal obstruction syndrome (SOS), and lower overall survival [5,6]. Going below the therapeutic range can cause graft failure and disease relapse [7]. Many attempts have been made to take into account the potential influencing factors, such as gender, age, weight, metabolism pathway and genetic profile. Unfortunately, the prediction of busulfan exposure still remains unachievable. Moreover, pediatric Asian patients are usually considered the most vulnerable patients in need due to the metabolism’s immaturity and the increasing incidence of toxicity in children [8,9], as well as the low minor allele frequency in Asians. This scarcity creates a burden on the patient’s clinical treatment, as well as on conducting proper genetic investigations in the Asian population compared to the Caucasian population [10,11].

There have been many studies on the metabolism of busulfan. Some studies proposed the involvement of glutathione *S*-transferase (GST) [12], whereas others indicated the involvement of cytochrome P450 (CYP) enzymes, such as *CYP2B6* and *CYP39A1* [13,14,15]. Unlike GST, clear biological evidence of the involvement of CYP enzymes is still absent; thus, busulfan cannot be considered their substrate. Furthermore, CYP enzymes are believed to participate in the formation of sulfolane, a metabolite of Bu, in the downstream oxidation period, but not the active parent molecule [12,13]. At our current level of knowledge, GST is believed to be a direct and the most predominant metabolizer of busulfan.

GST, an enzyme of phase II metabolism in liver cells encoded by the *GST* gene, has successfully been isolated in eight subfamilies [16], among which *GSTA1*, *GSTM1*, and *GSTP1* are the most paramount. In an vitro study, Czerwinski et al. [17] extracted GST enzymes from the liver cells of a female Caucasian donor and revealed that the activities of *GSTM1* and *GSTP1* enzymes are equal to around 46% and 18% of that of the *GSTA1* enzyme, respectively. The polymorphism of *GSTA1* essentially occurs in the promotor region. The variants of three linkage disequilibrium loci (567T > G, 69C > T, 52G > A) weaken the binding force of the Sp1 transcription factor on the promotor region and decrease the expression of the *GSTA1* enzyme [18]. The whole-gene deletion of *GSTM1* and the nonsynonymous variant at locus 313A > G of *GSTP1* can synthesize dysfunctional enzymes or completely prevent synthesis. These genetic impacts have been examined in Caucasians [19,20] and some adult Asians [10,21]. Pediatric Asians were also investigated by some authors [22] but the results remained negative, in contrast to Israelis [23]. The Israeli population is geographically considered Asian but their *GSTA1* genetic profile is more similar to Caucasians [23]. The reasons for this phenomenon in Asians may be due to the low minor allele frequency [22], which results in a burden on the sample size for investigation in Asians, and which may be due to the metabolism’s immaturity in children, causing greater fluctuation in their PK behaviors.

In consideration of the abovementioned drawbacks, this study was aimed at investigating the potential impact of *GSTA1*, *GSTM1* and *GSTP1* genetic polymorphisms on the PK properties of busulfan in pediatric Asian patients.

## 2. Patients and Methods

### 2.1. Patients and Conditioning Regimens

In this retrospective cohort study, we recruited 92 pediatric patients who underwent haploidentical HSCT in the PPM Laboratory, Ramathibodi Hospital, Mahidol University from September 2015 to September 2020. Before HSCT, patients received IV busulfan (Busulfex, Otsuka America Pharmaceutical, Rockville, MD, USA) once daily for 4 days. The first dose was personalized on the basis of body surface area (BSA) and age. Children < 2 years old, 2–6 years old and >6 years old were administered 80, 120 and 130 mg/m^2^/day of busulfan, respectively. Therapeutic drug monitoring (TDM) was performed to adjust dosages until the target range was attained on subsequent days. The Medical Ethics Committee of Ramathibodi hospital, Mahidol University approved the study (protocol code ID 04-61-37 and date of approval 12 June 2018). All patients and/or parents provided informed consent in accordance with the Helsinki Declaration.

### 2.2. Conditioning-Related Regimen and Prophylaxis of Infection

These regimens were covered before and after transplantation. During busulfan administration, patients were also prescribed ranitidine for gastroprotection, phenytoin for anti-epilepsy, and ciprofloxacin, penicillin V and acyclovir for prophylaxis of infection. Furthermore, fludarabine was administered 1 h before busulfan as part of the conditioning regimen.

### 2.3. DNA Extraction and GST Genotyping

Pretransplant genomic DNA was extracted using magnetic bead technology, which was executed by MagNA Pure Compact nucleic acid purification kit and MagNA Pure Compact Instrument (Roche Diagnostics Ltd.^®^, Indianapolis, IN, USA). The extracted DNA was then quantified by a NanoDrop Spectrophotometer (Thermo Scientific^®^, Waltham, MA, USA) at wavelength of 230, 260 and 280 nm. An extracted DNA concentration higher than 50 ng/µL is typically considered qualified enough. The TaqMan allelic discrimination method (Applied Biosystem^®^, Foster City, CA, USA) was used for the detection of the *GSTP1* gene (c.A313G; rs1695; pI105V), single-nucleotide polymorphisms (SNPs), and *GSTM1* copy number variations, as previously described [24]. The *GSTM1* and *GSTP1* genes were amplified by TaqMan genotyping assay (Applied Biosystems^®^, Waltham, MA, USA). Their catalog numbers are Hs02575461_cn and C___3237198_20, respectively. All real-time PCR plates were prepared as per the instructions of manufacturers and run inside a real-time PCR system Viia 7 (Life Technology^®^, Foster City, CA, USA). The operating software of real-time PCR system is QuantStudio version 1.3 (Applied Biosystems^®^, MA, USA). For *GSTM1*, Copy Caller Software version 2.1 (Applied Biosystems^®^, MA, USA) was additionally used to identify CNVs. In addition, Sanger sequencing (Applied Biosystems^®^, MA, USA) was performed as a genotyping service (U2Bio^®^, Bangkok, Thailand) to investigate the promoter region of *GSTA1* that defines *A and *B alleles (69C, -52G, designated as GSTA1*A; -69T, -52A, designated as GSTA1*B). Due to the distance between the two SNPs, C-69T (rs3957357) and G-52A (rs3957356), being very short (just 17 nucleotides), Sanger sequencing was the optimal method for their detection and determination of linkage disequilibrium in the Thai population. The promoter region of *GSTA1* gene was amplified with the forward primer *GSTA1*-F (5′–ACT TTG ATT GCC AAC CTT GAA–3′) and the reverse primer *GSTA1*-R (5′–TTA AAC GCT GTC ACC GTC CT–3′). The referring primers were commercially provided by manufacturer (Thermo Scientific^®^, MA, USA) and they worked follow the thermal program: 72 °C in 10 min, then 40 cycles of temperature (96 °C in 10 s, 61 °C in 15s, 72 °C in 20 s), and finally 72 °C in 10 min.

### 2.4. Pharmacokinetic Analysis

The TDM results of the first dose and second dose were used to evaluate the genetic impact. Blood samples were collected from peripheral veins to avoid contamination with IV busulfan in the central venous catheter. The selected timepoints were 0, 180, 195, 210, 240, 300, 360 and 480 min. Blood samples were maintained in EDTA at 4 °C before being quantified. Busulfan determination and validation was performed by an LC system (Agilent Technologies^®^, Santa Clara, CA, USA) and by a detector MS/MS (AB Sciex^®^, Vaughan, ON, Canada), as described elsewhere [25] with minor modifications. Internal standard (IS) is busulfan-d8 (CDN Isotopes^®^, Pointe-Claire, QC, Canada). Before being injected to the LC-MS/MS system, busulfan was extracted by Solid Phase Extraction (SPE) cartridges (Waters^®^, MA, USA). Briefly described, 520 µL sample (200 µL plasma sample, 20 µL IS and 300 µL deionized water) was loaded on SPE instrument (Waters^®^, Milford, MA, USA). Busulfan was then retrieved from SPE cartridges by methanol (RCI Labscan^®^, Bangkok, Thailand) and injected into LC-MS/MS system. The retention time of busulfan and IS were around 5 and 5.15 min, respectively. Each patch run had quality control at 3 concentrations (600, 2700, 4800 ng/mL). Linear range was from 200 to 6000 ng/mL, built from 7-point calibration. Linear regression constant (R^2^) was higher than 0.999 in all validated batches. The accuracy and precision of the lowest point (200 ng/mL) was varied within ±20%. The accuracy and precision of 6 other points was varied within ±15%. PK analysis was performed using the noncompartmental model of PKanalix version 2020R1 (Lixoft^®^, Paris, France). The following PK parameters were estimated: half-life (t_1__/2_), clearance (CL), highest concentration (C_max_), volume of distribution (Vd), and area under the concentration–time curve (AUC).

### 2.5. Statistical Analysis

*t*-tests and ANOVA tests were employed to evaluate the PK differences where appropriate. All analyses and data visualization were performed by R software version 4.0.2 (R Foundation for Statistical Computing, Vienna, Austria). Results were considered significant at *p* < 0.05.

## 3. Results

### 3.1. Patient Characteristics

Table 1 summarizes the demographic information of the 92 patients. Their mean age was 8.4 ± 5.12 years old, and their mean weight was 26.52 ± 14.75 kg. Out of 92, 40 patients had thalassemia. All patients received IV busulfan once daily prior to haploidentical hematopoietic stem-cell transplantation. All patients received phenytoin as part of their anti-seizure regimen. Fludarabine was administered concomitantly with busulfan in 72 patients. The PK differences were not statistically significant between thalassemia and non-thalassemia patients, nor between patients with and without fludarabine.

### 3.2. Frequencies of *GSTA1*, *GSTM1* and *GSTP1* Genotypes

The allele frequencies of *GSTA1*, *GSTM1* and *GSTP1* are illustrated in Table 2. All detected SNPs were within the Hardy–Weinberg equilibrium (*p* > 0.05). For *GSTM1*, deletions and 1–3 copy number variations (CNVs) were detected, and the tests for Hardy–Weinberg equilibrium were not performed because our genotyping technique cannot distinguish if an individual with 2 CNVs is homozygous GSTM1*1CNV/*1CNV or heterozygous GSTM1*2CNV/*deletion [26,27]. The frequencies of GSTA1*B, GSTM1*null/*null, and GSTP1*G were 16.9%, 68.5%, and 21.2%, respectively.

### 3.3. Overview of Genetic Influences on PK of Busulfan

Table 3 provides an overview of the associations of GST genetic polymorphisms with each PK parameter. These differences were seen more clearly in the first dose than in the second dose. In both doses, statistically significant differences in PK parameters were observed in patients with GSTA1*B, but not in patients with GSTP1*A and the *GSTM1* deletion. These differences were noted in terms of t, CL, C_max_ and AUC for the first dose and t_1__/2_ for the second dose. Compared to noncarriers of the variant GSTA1*B, carriers of it had a higher t_1__/2_ (for first dose—161.9 vs. 134.3 min, *p* = 0.0016; for second dose—156.1 vs. 129.8, *p* = 0.012), lower CL (88.74 vs. 124.23 mL/min, *p* = 0.0089), higher C_max_ (4232.6 vs. 3675.5 ng/mL, *p* = 0.0021), and higher AUC (5310.6 vs. 4177.1 µM/min, *p* = 0.00033).

### 3.4. The Impact of GSTA1 Polymorphism on PK of Busulfan

**Figure 1:**
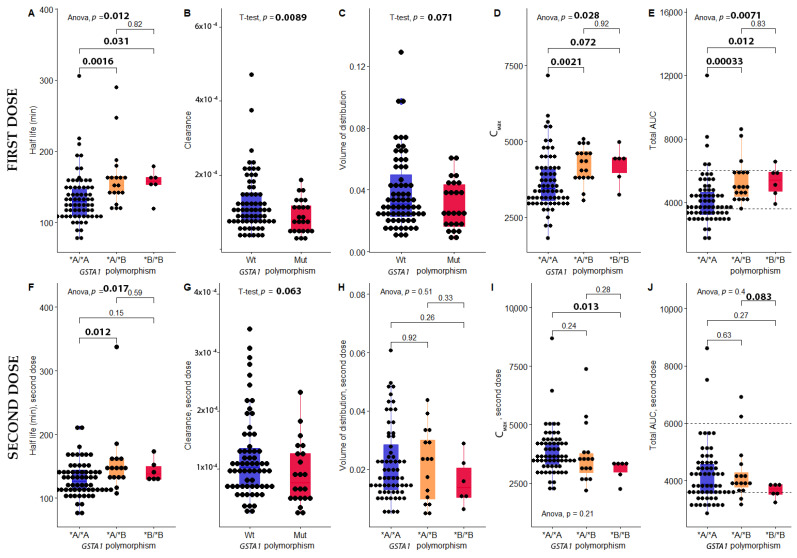
The impact of *GSTA1* polymorphism on each PK parameter of Busulfan at fist dose (**A**): Half-life; (**B**): Clearance; (**C**): Volume of distribution; (**D**): C_max_; (**E**): Total AUC, and at second dose: (**F**): Half-life; (**G**): Clearance; (**H**): Volume of distribution; (**I**): C_max_; (**J**): Total AUC. *GSTA1*: Glutathione-S-tranferase; AUC: Area Under the Curve; Wt: Wild-type; Mut: Mutant; *A/*A: Homozygous wild-type; *A/*B: Heterozygous; *B/*B: Homozygous mutant. Anova: Compare more than 2 groups, 2 tails *t*-test: Compare 2 groups. Statistically significant threshold: 0.05.

Figure 1 describes the statistically significant impacts of *GSTA1* polymorphism on the PK of the first and second doses of busulfan. These impacts were detected more clearly in the first dose than in the second dose. For the analysis of CL of both doses and the analysis of Vd of the first dose, patients were classified into two groups, wild-type and variant, due to the small sample size. For other analyses, patients were classified into three groups: homozygous wild-type, heterozygous, and homozygous mutant. In terms of the PK of the first dose (before adjustment), the carriers of variant allele GSTA1*B had a higher half-life, a higher C_max_, a higher AUC_0__–inf_, a lower CL, and a suggestive trend of a lower Vd (*p* = 0.071). In terms of the PK of the second dose (after adjustment), *GSTA1* created a difference in the half-life, along with a suggestive trend of difference in the CL (*p* = 0.063).

Before dose adjustment, the percentage of patients higher than, within, and lower than the target range was 11%, 59% and 30%, respectively (Figure 1E). After dose adjustment, these numbers were 5%, 74% and 21%, respectively (Figure 1J). The mean AUCs of homozygous wild-type, heterozygous variant, and homozygous variant of *GSTA1* were 4177, 5310, and 5296 µM/min, respectively (Figure 1E). The carriers of variant GSTA1*B exhibited an augmentation in AUC of around 27.1% (*p* = 0.0071).

### 3.5. Multivariate Regression Analysis between PK of Bu and Three Independent Variables: BSA, GSTA1 and Gender

The multivariable regression analysis in Table S2 illustrates that the PK of Bu was impacted by BSA, *GSTA1* polymorphism and gender. Through each step of inclusion of the new variable, the new model was compared with the previous one, whereby the new one showed a better performance in terms of fitting (F statistic and ∆F with *p* < 0.05). 

In the group of all patients, BSA and *GSTA1* polymorphism had impacts on t_1__/2_ and CL, whereas BSA, *GSTA1* polymorphism and gender had impacts on Vd. After stratification of patients at the age of 6, BSA and *GSTA1* polymorphism became the first and second most important factors for those below the threshold, whereas *GSTA1* polymorphism was the most important factor for those above the threshold. 

## 4. Discussion

Among all pediatric Asian populations, this study is the first to successfully detect the genetic impacts on the PK disposition of busulfan. More specifically, we targeted the Thai pediatric population expected to receive HSCT. The genetic impacts of this specific population remained unknown until today because (1) children’s metabolism varies widely across individuals, and (2) there is a lower allele frequency in Asians than in Caucasians [10], with the exception of Israelis. However, this genetic culprit in Asians is still prevalent enough to make this specific population vulnerable. This study provides evidence that the *GSTA1* polymorphism has an impact on the PK of busulfan.

In our cohort, phenytoin and ciprofloxacin were administered to all patients. Therefore, the potential confounding effects of drug–drug interactions between phenytoin [28,29] or ciprofloxacin and busulfan were minimized. The distributions of allele frequencies differ across ethnicities. Regarding *GSTA1*, the current analysis reported the frequency of GSTA1*B at around 16.9% which is 2–3 times lower than reported in Caucasians [19,30,31,32,33,34], but similar to other Asian populations, such as the Chinese [21,35,36], Koreans [37], and Japanese [22,38]. Two SNPs detected in *GSTA1*, G-52A (rs3957356) and C-69T (rs3957357), are in a full linkage disequilibrium, in line with other populations, such as Caucasians [19,34], Koreans [30] and Chinese [21]. The polymorphisms of *GSTM1* included deletions and 1–3 CNVs in our cohort. It was infeasible to test for the Hardy–Weinberg equilibrium of *GSTM1* using real-time PCR genotyping. A genotyping method with higher resolution such as sequencing should be adopted to identify the exact positions of each *GSTM1* sequence on its homologous chromosomes. In this study, GSTP1*G had a frequency of 21.2%, which is consistent with other Asian populations [11,21], but lower than observed in Caucasians (~37–45%) [19,32,33].

The adjustment of individual doses hindered the detection of genetic impacts in second doses with respect to first doses. Hence, in the first dose, the genetic impacts of *GSTA1* were related to half-life, C_max_, AUC, CL and Vd, whereas, in the second doses, only half-life and CL registered impacts with *p*-values of 0.017 and 0.063, respectively. The carriers of the variant allele GSTA1*B had a lower CL, which in turn reduced the elimination of busulfan; this was associated with increased AUC. In contrast to *GSTA1*, we did not detect any statistical significance in *GSTM1* and *GSTP1*. This observation can be explained by the quantity of *GSTM1* and *GSTP1* enzymes in liver cells (equal to 46% and 18% of *GSTA1*, respectively) [17]. Nishikawa et al. [22] investigated Japanese pediatric patients but obtained negative results due to the small sample size, which reduced the power of detection. In Israeli pediatric patients, Elhasid et al. [23] successfully proved the impact of *GSTA1* on the PK of busulfan. Even though Israel is geographically considered to be a part of Asia, the allele frequency of GSTA1*B in Israelis is 55%, which is similar to some Caucasian populations [15,19,39], and far higher than in other Asian populations [10,22]. The reason for the similarity between Israelis and Caucasians can be explained by geography and history. Indeed, in Caucasian pediatric populations, many authors found an effect of *GSTA1*. Ansari et al. [19] conducted a meticulous study using a multicenter cohort and proved the associations of *GSTA1* and *GSTM1* not only with CL and AUC, but also with clinical outcomes such as sinusoidal obstruction syndrome (SOS), graft-versus-host disease (GvHD) and hemorrhagic cystitis (HC). Ten Brink et al. [15] provided evidence of *GSTA1* and *CYP39A1* polymorphisms as influencing factors of busulfan clearance. In contrast to *GSTA1*, a plausible explanation for the role of *CYP39A1* in busulfan metabolism is still lacking. More clarification is needed to establish a thorough understanding of the role played by *CYP39A1*. In adults, many authors confirmed the impacts of *GSTA1* in Asians and Caucasians (Supplementary Table S1), such as Kim et al. in Koreans [30], Terakura et al. in Japanese [14], and Michaud et al. in Canadians [40]. 

Even though the allele frequency of GSTA1*B in Asians is far lower than in Caucasians [10], this study suggests the incorporation of the *GSTA1* polymorphism into the dosing regimen of busulfan in pediatric Asians. With an allele frequency of ~17%, carriers of the GSTA1*B allele had an AUC augmentation of around 27.1%. Therefore, the adjusted dosages should be decreased accordingly in patients carrying GSTA1*B variant. Overexposure to busulfan may lead to GvHD, SOS, or lower overall survival [5,6]. Busulfan treatment in pediatric Asians can be more personalized if pharmacogenetic testing of *GSTA1* is implemented to adjust the doses accordingly.

The effect of nongenetic factors is given special attention in pediatrics. In our study, an involvement of BSA and gender was found. Table S2 shows that BSA was important in the group <6 years old. In contrast, BSA was not statistically significant in the older group, whereas *GSTA1* polymorphism emerged as important. The inconsistency between the two groups can be explained by the maturation of liver enzymes along with the growth of BSA [41]. The impact of gender on activity of the microsomal GST enzyme has been suggested in mice [42] and humans [43]. This impact can be explained by the participation of testosterone/estrogen [44,45,46], in the expression of the microsomal GST enzyme. The impacts of nongenetic factors were also noted in pediatric Caucasians. Ansari et al. [19] described the effects of nongenetic factors age and gender, whereas Ten Brink et al. [15] found an effect of age but not gender. Interestingly, Nava et al. [39] defined the maturation level of children’s enzymes using another parameter, Fmat. This parameter was shown to be associated with the PK of Bu. Even though many nongenetic factors have been reported, most of these factors represent the maturation of liver enzymes.

This research had some limitations, necessitating future efforts to clarify several issues. First, this study classified patients into carriers and noncarriers. However, upon increasing the sample size, patients can be further classified into subgroups, as suggested by Ansari et al. [19], taking into account more genetic variants to achieve more personalized treatment. Second, this study included children <6 years old. These infants and toddlers are more fragile and pharmacokinetically unpredictable from a clinical point of view. Thus, more analyses should be performed in these age groups. Third, the association between *GST* polymorphism and clinical outcomes was not assessed in the current study, which could produce more persuasive evidence of the genetic impacts on Bu response. Lastly, the role of drug–drug interactions should be taken into consideration in further studies, such as between fludarabine and busulfan [47,48], between cyclophosphamide and busulfan [49,50], between phenytoin and busulfan [28,29], and between ciprofloxacin and busulfan [51].

In conclusion, *GSTA1* polymorphisms were significantly associated with variations in the PK profile of busulfan and could be considered potential pharmacogenetic factors when personalizing busulfan treatment in pediatric Asians. The findings of this study may accelerate the implementation of precision medicine when administering busulfan in clinical practice. *GSTM1* and *GSTP1* polymorphisms were not found to be statistically significant factors in this study; however, more research is needed to properly understand their role in pediatric Asians receiving busulfan.

## Figures and Tables

**Table 1:** Patient characteristics.

Characteristics		Number of Patients	Percentage
Gender	Male	48	53%
	Female	44	47%
Diagnosis	Thalassemia	40	43.5%
	Non-thalassemia	52	56.5%
	Neuroblastoma	9	9.9%
	AML	7	7.6%
	ALL	8	8.6%
	CML	1	1.1%
	JMML	1	1.1%
	Metabolic diseases	3	3.3%
	Immunodeficiencies	14	15%
	AIHA	1	1.1%
	MDS	2	2.2%
	SAA	3	3.3%
	Osteopetrosis	1	1.1%
	Undifferenciated round-cell tumor	1	1.1%
	Other	1	1.1%
Conditioning regimen	BuCyMesna	7	7.6%
	BuCyATGMesna	1	1.1%
	BuMel	4	4.3%
	BuMelATG	4	4.3%
	BuFluATG	46	49.9%
	BuFluThio	19	20.7%
	BuFluCyATGMesna	1	1.1%
	BuFluMelATG	3	3.3%
	BuFluATGRit	3	3.3%
	BuFluThioRit	2	2.2%
	Bu and BuCyATG	2	2.2%
Age	0–2 years old	8	8.7%
	2–6 years old	27	29.3%
	6–21 years old	57	62%
Age (year)	Min	0.42	
	Mean	8.41	
	Max	21.59	
BSA (square meters)	Min	0.27	
	Mean	0.935	
	Max	1.83	
Height (centimeter)	Min	53.40	
	Mean	121.91	
	Max	175.0	
Weight (kilograms)	Min	4.10	
	Mean	26.52	
	Max	72.20	

AML, acute myeloblastic leukemia; ALL, acute lymphoblastic leukemia; CML, chronic myeloblastic leukemia; JMML: juvenile myelomonocytic leukemia; AIHA, autoimmune hemolytic anemia; MDS, myelodysplastic syndrome; SAA, severe aplastic anemia; Bu, busulfan; Mel, melphalan; Cy, cyclophosphamide; Flu, fludarabine; ATG, anti-thymocyte globulin; Thio, thiotepa; Rix, rituximab; Mesna, 2-mercaptoethane sodium sulfonate.

**Table 2:** Distributions of *GSTA1*, *GSTM1* and *GSTP1* variants in all patients.

Gene	Variants		*N* (%)	HWE
*GSTA1*	Diplotype	* *A* /* *A*	67 (72.8%)	0.2542
		* *A* /* *B*	19 (20.7%)	
		* *B* /* *B*	6 (6.5%)	
	Haplotype	*A	83.1%	
		*B	16.9%	
*GSTP1*	Diplotype	*A/A*	55 (59.8%)	0.5896
		*A* */* *G*	35 (38.0%)	
		*G/G*	2 (2.2%)	
	Haplotype	* *A*	78.8%	
		* *G*	21.2%	
*GSTM1*	*Deletion*		63 (68.4%)	NA
	*CNV* *:* *1*		26 (28.3%)	
	*CNV* *:* *2*		2 (2.2%)	
	*CNV* *:* *3*		1 (1.1%)	

For *GSTA1*, two detected SNPs (G-52 (rs3957656) and C-69T (rs3957657)) were in a full linkage disequilibrium: CNV—copy number variation; NA—not applicable; HWE—Hardy-Weinberg equilibirum.

**Table 3:** Differences between pharmacokinetic parameters of Bu and genetic polymorphisms (*t*-test or ANOVA).

First Dose					
Polymorphism	t_1__/__2_	CL	V_d_	C_max_	AUC_0__–__inf_
*GSTA1*					
*A/*A (*n* = 67)	134.34 ± 35.71 ^Ŧ^	124.23 ± 78.33 *	23,434.0 ± 14,292.0	3675.5 ± 949.4 ^Ŧ^	4177.1 ± 1557.9 ^Ŧ^
*A/*B (*n* = 19)	161.86 ± 42.61 ^Ŧ^	94.01 ± 47.40 *	21,896.3 ± 11,570.5	4232.6 ± 581.9 ^Ŧ^	5310.6 ± 1347.1 ^Ŧ^
*B/*B (*n* = 6)	154.99 ± 19.96 ^Ŧ^	72.05 ± 37.36 *	16,266.7 ± 9879.6	4231.7 ± 605.9 ^Ŧ^	5296.4 ± 968.6 ^Ŧ^
***GSTM1***					
Deletion (*n* = 63)	142.13 ± 42.09	119.75 ± 65.23	23,703.8 ± 12,622.1	3787.1 ± 849.8	4476.1 ± 1698.7
1 *CNV* (*n* = 26)	141.27 ± 27.75	105.37 ± 90.54	20,971.9 ± 16,022.6	3896.9 ± 1048.9	4471.6 ± 1250.5
2 or 3 *CNV* (*n* = 3)	126.16 ± 26.63	85.90 ± 34.80	15,033.3 ± 5533.8	4053.3 ± 447.7	4743.6 ± 1146.0
***GSTP1***					
*A/A* (*n* = 55)	142.92 ± 36.82	115.71 ± 77.39	23,020.2 ± 14,464	3848.5 ± 956.7	4504.8 ± 1654.8
*A/G* (*n* = 35)	139.91 ± 41.03	114.48 ± 65.27	22,491.7 ± 12,157.1	3793.4 ± 832.6	4460.9 ± 1463.5
*G/G* (*n* = 2)	124.21 ± 0.93	85.20 ± 85.98	15,195 ± 15,280.6	3815 ± 49.5	4324.5 ± 201.9
**Second Dose**					
**Polymorphism**	**t_1_**_/_ **_2_**	**CL**	**V_d_**	**C_max_**	**AUC_0_**_–_ **_inf_**
***GSTA1***					
*A/*A (*n* = 64)	129.81 ± 27.29 ^Ŧ^	119.31 ± 70.74	22,007.0 ± 12,898.1	3825.5 ± 968.6	4165.1 ± 1005.7
*A/*B (*n* = 16)	156.09 ± 52.20 ^Ŧ^	97.29 ± 59.73	21,496.3 ± 12,524.5	3679.4 ± 1290.5	4265.8 ± 998.4
*B/*B (*n* = 6)	142.65 ± 17.61 ^Ŧ^	75.85 ± 38.68	15,775 ± 8377.1	3070 ± 446.1	3646.5 ± 257.7
***GSTM1***					
Deletion (*n* = 60)	137.17 ± 38.34	117.83 ± 66.28	22,707.5 ± 12,469.3	3781 ± 1090.0	4186.4 ± 1003.3
1 *CNV* (*n* = 23)	129.50 ± 20.51	101.40 ± 74.80	18,743.0 ± 13,203.6	3715.2 ± 883.8	4088.3 ± 966.9
2 or 3 *CNV* (*n* = 3)	150.70 ± 20.11	81.83 ± 25.72	17,833.3 ± 6560.7	3270 ± 350	3827.8 ± 392.1
***GSTP1***					
*A/A* (*n* = 52)	134.27 ± 25.37	116.27 ± 73.34	21,873.6 ± 12,824.2	3643.3 ± 751.2	4053.0 ± 808.3
*A/G* (*n* = 32)	138.65 ± 45.70	106.94 ± 58.18	21,238.8 ± 12,294.6	3807.8 ± 1213.3	4260.7 ± 1158.8
*G/G* (*n* = 2)	121.12 ± 10.97	89.95 ± 94.82	14,980 ± 15,160.3	5410 ± 2771.9	4800.1 ± 2017.4

* Statistically significant according to two-tailed *t*-test analysis (*p* < 0.05) between group 1 homozygous wildtype (or deletion), group 2 heterozygous (or 1 *CNV*), and group 3 homozygous variant (or 2 or 3 *CNV*); Ŧ Statistically significant according to one-way ANOVA (*p* < 0.05) between group 1 homozygous wildtype (or deletion), group 2 heterozygous (or 1 *CNV*), and group 3 homozygous variant (or 2 or 3 *CNV*). The units of PK parameters: t_1__/2_, min; CL, mL/min; Vd, mL; C_max_, ng/mL; AUC_0__–inf_, µM/min.

## Data Availability

The data that support the findings of this study are available from the corresponding author upon reasonable request.

## Supplementary Materials

The following are available online at https://www.mdpi.com/article/10.3390/pharmaceutics14020401/s1: Table S1. Review of 18 articles regarding polymorphisms of the *GST* family and the PK of Busulfan [52,53,54,55]; Table S2. Multivariate regression analysis between PK of Bu and three independent variables: BSA, *GSTA1,* and gender.
