# Supplementary material for: Effect of GSTA1 Variants on Busulfan-Based Conditioning Regimen Prior to Allogenic Hematopoietic Stem-Cell Transplantation in Pediatric Asians
Source: Pharmaceutics. 2022 Feb 11;14(2):401. doi: 10.3390/pharmaceutics14020401 (PMC8880478; doi:10.3390/pharmaceutics14020401)
Supplement: Supplementary file 1 [file pharmaceutics-14-00401-s001.zip › pharmaceutics-1524073-supplementary.pdf]

pharmaceutics

Supplementary Materials

Effect of *GSTA1* Variants on Busulfan-Based Conditioning Regimen Prior to Allogenic Hematopoietic Stem-Cell Transplantation in Pediatric Asians

Ai-Hoc Nguyen [1,2], Mohitosh Biswas [1,2,3], Apichaya Puangpetch [1,2], Santirhat Prommas [1,2], Samart Pakakasama [4], Usanarat Anurathapan [4], Jiratha Rachanakul [1,2], Rattanaporn Sukprasong [1,2], Nutthan Nuntharadtanaphong [1,2], Nutcha Jongjitsook [1,2], Suradej Hongeng [4] and Chonlaphat Sukasem [1,2,5,6,*]

[1] Division of Pharmacogenomics and Personalized Medicine, Department of Pathology, Faculty of Medicine Ramathibodi Hospital, Mahidol University, Bangkok 10400, Thailand; aihoc050994@gmail.com (A.-H.N.); biswas_07pharm@ru.ac.bd (M.B.); apichaya.pua@mahidol.ac.th (A.P.); Santirhat.pro@mahidol.ac.th (S.P.); jiratha.rac@mahidol.edu (J.R.); rattanaporn.suk@mahidol.ac.th (R.S.); nutthan.nun@mahidol.ac.th (N.N.); PearNutcha_1997@hotmail.com (N.J.)
[2] Laboratory for Pharmacogenomics, Somdech Phra Debaratana Medical Center (SDMC), Ramathibodi Hospital, Bangkok 10400, Thailand
[3] Department of Pharmacy, Faculty of Science, University of Rajshahi, Rajshahi 6205, Bangladesh
[4] Division of Hematology-Oncology, Department of Pediatrics, Faculty of Medicine Ramathibodi Hospital, Mahidol University, Bangkok 10400, Thailand; samart.pak@mahidol.ac.th (S.P.); usanarat.anu@mahidol.ac.th (U.A.); suradej.hon@mahidol.ac.th (S.H.)
[5] Division of Pharmacogenomics and Precision Medicine, The Preventive Genomics & Family Check-Up Services Center, Bumrungrad International Hospital, Bangkok 10400, Thailand
[6] MRC Centre for Drug Safety Science, Department of Pharmacology and Therapeutics, Institute of Systems, Molecular and Integrative Biology, University of Liverpool, Liverpool L69 3GL, UK
* Correspondence: chonlaphat.suk@mahidol.ac.th

Table S1. Review of 18 articles about polymorphism of GST family and PK of busulfan.

		Authors	Sample Size	Ethnicity	Associated PK Parameters	Associated Genetic Markers	References
Pediatrics	Asian	Nishikawa et al.	20	Japanese	Not detected	Not detected	[22]
		Elhasid et al.	18	Israeli	AUC; Cmax; Cl	*GSTA1; GSTM1; GSTP1.*	[23]
	Caucasian	Ansari et al.	138	Caucasian; African	AUC; Cl;	*GSTA1; GSTM1;*	[19]
		Nava et al.	112	Caucasian; African	Cl; Vd.	*GSTA1;*	[39]
		Ansari et al.	44	Middle-east	AUC; Css; Cmax; Cl	*GSTA1;*	[24]
		Ten Brink et al.	84	Caucasian	Cl;	*GSTA1; CYP39A1;*	[15]
		Nava et al.	101	Caucasian	Css; Cl; AUC	*GSTA1;*	[52]
Adults	Asian	Terakura et al.	55	Japanese	AUC	*GSTA1;*	[14]
		Yin et al.	25	Chinese	AUC; Cl; Cmax; Vd; T1/2	*GSTA1; GSTP1*	[21]
		Choi et al.	36	Korean	AUC; Cl; Vd	*GSTA1*	[37]

	Kusama et al.	12	Japanese	Ke; Cl/F; C_{mean};	*GSTA1*	[53]
	Sun et al.	43	Chinese	Not detected	Not detected	[10]
	Kim et al.	58	Korean	Cl	*GSTA1;* Combination of GSTM1/GSTT1	[30]
Caucasian	Michaud et al.	87	Canadians	AUC; Cl/F	*GSTA1*	[40]
	Marloes et al.	62	Dutch	Cl	*GSTA1;*	[54]
	Ten Brink et al.	66	Dutch	Cl, AUC	*GSTA1*	[55]
	Bremer et al.	114	Norwegian	Css	*GSTA1*	[33]
	Abbasi et al	151	Caucasian	Cl	*GSTA1*	[34]

Vd: Volume of distribution; Cl: Clearance; AUC: Area Under the Curve; PK: Pharmacokinetic; Ke:Elimination constant; Cl/F: Clearance corrected by bioavailability ; C_{mean}: Mean of concentration.

Table S2. Multivariate regression analysis between PK of Bu and three independent variables: BSA, *GSTA1* and gender.

Group	Step	Dependent Variables	Constant	BSA	Non-Carrier of *GSTA1*B*	Gender of Male	F Statistic	ΔF
All patients	1	$T_{1/2}$	118.68	24.27*	-	-	5.02**	-
	2	$T_{1/2}$	137.259	24.82*	−26.215**	-	7.739***	9.958***
	1	Cl	-3.358×10^{-5}	1.585×10^{-4}***	-	-	146.5***	-
	2	Cl	-5.720×10^{-5}	1.578×10^{-4}***	3.333×10^{-5}***	-	87.11***	11.149***
	1	Vd	−0.009	0.034***	-	-	425.9***	-
	2	Vd	−0.01	0.033***	-	0.03**	223.7***	4.564**
	3	Vd	−0.012	0.033***	0.003***	0.003**	156.5***	4.504**
≤6 years old	1	Cl	-1.782×10^{-5}	1.448×10^{-4}**	-	-	50.8***	-
	2	Cl	-4.029×10^{-5}	1.525×10^{-4}***	2.605×10^{-5}***	-	36.94***	9.695***
	1	Vd	−0.009	0.037***	-	-	148.6***	-
	2	Vd	−0.012	0.038***	0.003**	-	86.96***	5.417**
	3	Vd	−0.012	0.037***	0.003**	0.003**	66.14***	4.651**
>6 years old	1	$T_{1/2}$	169.963	-	−33.181***		9.625***	-
	2	$T_{1/2}$	158.874	-	−30.035***	15.525*	6.372***	2.803*
	1	Cl	1.128×10^{-4}	-	3.903×10^{-5}*	-	3.033*	-

*: p value < 0.1; **: p value < 0.05; ***: p value < 0.01.
